# Moisture Content Prediction in Polymer Composites Using Machine Learning Techniques

**DOI:** 10.3390/polym14204403

**Published:** 2022-10-18

**Authors:** Partha Pratim Das, Monjur Morshed Rabby, Vamsee Vadlamudi, Rassel Raihan

**Affiliations:** 1 Institute for Predictive Performance Methodologies, The University of Texas at Arlington Research Institute, Fort Worth, TX 76118, USA; 2 Mechanical and Aerospace Engineering Department, The University of Texas at Arlington, Arlington, TX 76019, USA

**Keywords:** FRP composites, dielectric analysis, moisture absorption, machine learning

## Abstract

The principal objective of this study is to employ non-destructive broadband dielectric spectroscopy/impedance spectroscopy and machine learning techniques to estimate the moisture content in FRP composites under hygrothermal aging. Here, classification and regression machine learning models that can accurately predict the current moisture saturation state are developed using the frequency domain dielectric response of the composite, in conjunction with the time domain hygrothermal aging effect. First, to categorize the composites based on the present state of the absorbed moisture supervised classification learning models (i.e., quadratic discriminant analysis (QDA), support vector machine (SVM), and artificial neural network-based multilayer perceptron (MLP) classifier) have been developed. Later, to accurately estimate the relative moisture absorption from the dielectric data, supervised regression models (i.e., multiple linear regression (MLR), decision tree regression (DTR), and multi-layer perceptron (MLP) regression) have been developed, which can effectively estimate the relative moisture absorption from the dielectric response of the material with an R¬2 value greater than 0.95. The physics behind the hygrothermal aging of the composites has then been interpreted by comparing the model attributes to see which characteristics most strongly influence the predictions.

## 1. Introduction

Fiber-reinforced polymer (FRP) composites’ endless utilization in basic applications of the aerospace industry, marine industry, civil infrastructures, and medical and energy sectors has been sprouting, due to their high strength-to-weight ratio and ease of application [1,2]. Based on the application, engineers have been able to modify the FRP composites’ mechanical properties by altering the manufacturing design and orientation of their principal components. However, FRP composites are susceptible to different internal damages if exposed to certain environmental conditions, such as moisture, high temperature, UV, etc. [3,4]. For example, increasingly, in the aviation sector, most of the crucial parts of the aircraft are made from FRP composites [5], which, in their service life, are exposed to varied moisture exposure. In the marine sector, vehicles are made from glass fiber-reinforced polymer composites, which encounter water continuously and degrade the material in a follow-through. The adverse effect of these water interactions with composites requires intensive study, and feasible techniques must be developed to detect these phenomena for maintenance purposes.

Moisture penetration depends primarily on the materials’ exposure period, according to various studies performed on cured polymers and FRP composites. Fick’s law of mass diffusion [6] theoretically explains this behavior. According to Fickian theory, the absorption rate is high at the beginning of moisture exposure, progressively drops, and ultimately approaches saturation when the material can no longer absorb any more moisture. Not all composites, however, follow the Fickian principle; instead, they tend to follow unique patterns, depending on the constituents [7]. Moisture diffusion follows a similar pattern in most FRP composites, except that the moisture content in the material continues to rise, but at a slower pace. Pseudo-Fickian conduct is what this is known as. However, moisture absorption in composites is a complex phenomenon that also depends on other factors, including the material system, type and nature of the matrix and fiber, fiber morphology, etc. [8].

**Figure 1:**
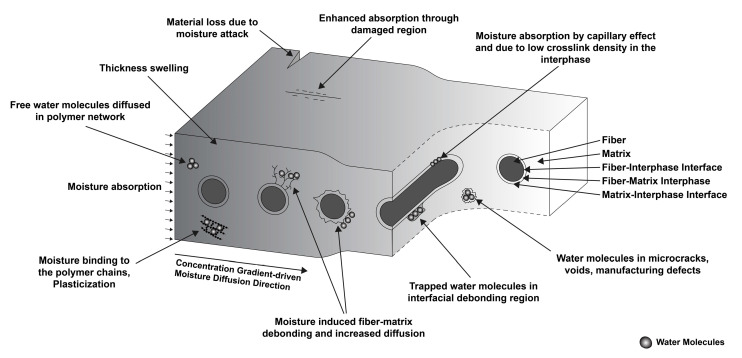
Schematic summary of different states of water molecules absorbed in FRP composites.

In essence, the water molecules inside composite materials can remain in two different forms—free or bound. Free water molecules remain in the polymer matrix between cracks, voids, or manufacturing defects. The fiber–matrix interfacial region can also hold free water molecules. These trapped water molecules can eventually be released if the desorption conditions are met for the composite [9]. However, bound water molecules are chemically trapped in the matrix. In FRP composites, the matrix consists of a resin (i.e., epoxy, etc.), hardener (i.e., diamine, etc.), and other chemical constituents, depending on the manufacturer [10]. These chemical compounds primarily consist of, or can react to, hydrophilic hydroxyl groups (-OH) or amine groups (-NH_2_), which attract polar water molecules and create a hydrogen bond to trap the water molecules in the matrix [11]. Typically, in FRP composites, the absorbed moisture amount is meager. Still, the corresponding adverse effects are significant enough to cause different reversible and irreversible chemical changes, such as hydrolysis, chain scission, oxidation, plasticization, micro-crack development, interfacial debonding, etc. [12]. With desorption, the bound water molecules may or may not reside in the matrix, but decreased mechanical properties cannot be restored fully [13]. Different absorption mechanisms contribute to the overall absorption scenario, including diffusion, capillary effect, absorption through microcracks, manufacturing defects, fiber–matrix interface, etc. Figure 1 shows the different moisture absorption mechanisms evident in FRP composites.

In academia and industrial research, different experimental techniques have been used to monitor the moisture absorption behavior in FRP composites. The gravimetric approach (GA) is one of the most prevalent techniques used for this purpose [14]. This method has been used in almost all of the research carried out on the moisture absorption of composites. However, this method is not applicable for structural health monitoring, as it is only viable in laboratory-based studies. Other methods, including differential scanning calorimetry (DSC), Fourier transform infrared (FTIR), etc., have also been utilized in composite structures to detect the presence of moisture in composites [15]. However, these all have their limitations [16]. For instance, DSC tests require a very small size of samples that may not represent the actual moisture state of a composite, and this is not the non-destructive testing (NDT) method. FTIR, though an NDT, also focuses on a small sample area and is often limited to a minimal depth of the sample under consideration. 

**Figure 2:**
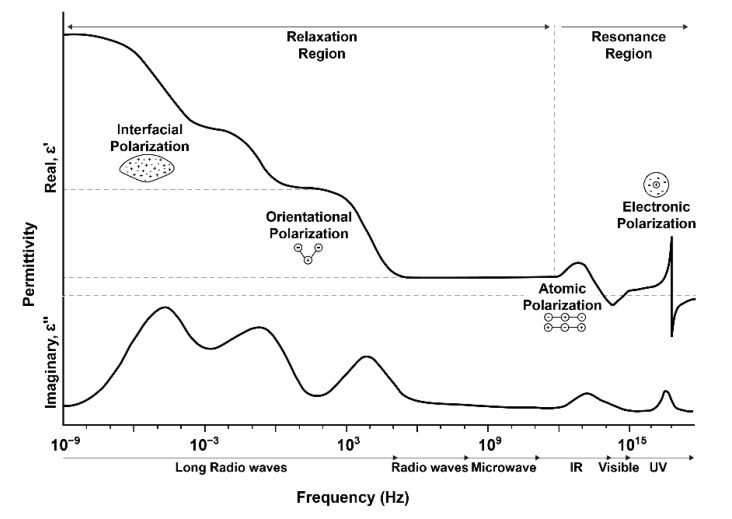
Different kinds of dielectric polarizations over broadband frequency range.

With technology advancements, different NDT methods are being introduced that focus on a different aspect of the material’s behavior to detect and monitor damages and contribute towards a predictive maintenance approach [17,18]. Impedance/dielectric spectroscopy is an NDE method that has been used in numerous sectors of structural health monitoring, including material state characterization [19,20], polymer cure monitoring [21], prepreg degradation [22,23], adhesive bonds [24,25], damage detection [26], etc. Broadband dielectric spectroscopy (BbDS) is another practical approach for studying the interaction of electromagnetic waves with materials at frequencies spanning from 10^−6^ to 10^12^ Hz. In the form of various dielectric characteristics of the material under investigation, this dynamic range can give information regarding molecular and dipolar perturbations, as well as charge transport and polarization effects, as described in Figure 2. As water molecules are dipoles in nature and create hydrogen bonds with the matrix component’s hydrophilic groups, they alter the composite’s dielectric properties by imposing different polarizations. This behavior can be detected, in terms of minute changes in dielectric properties, using IS/BbDS.

**Figure 3:**
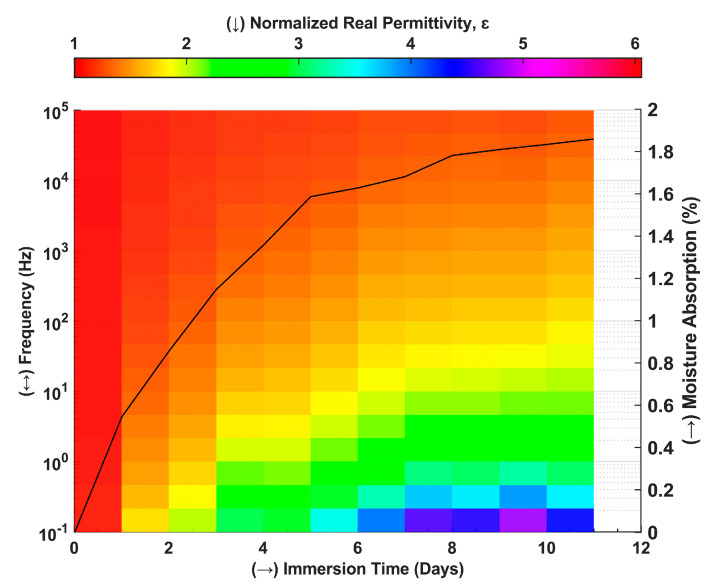
Heatmap of normalized real permittivity and trend of moisture absorption with immersion time [16].

Das et al. used BBDS to qualitatively characterize the moisture absorption-caused mechanical strength degradation in GFRP composites [16]. Figure 3 summarizes the results of the referenced work, where it is evident that the change in real permittivity strongly correlates with absorbed moisture with immersion time. This effect is most prominent in the lower frequency region, indicating ionic and interfacial polarization involvement. This explains the BBDS technique’s effectivity in qualitatively determining a composite’s current moisture state. However, these findings are only a proof of concept. A data-driven approach is required to explain this correlation further, where a prediction framework would be created to predict the specific moisture state in a composite in the service.

Recent advances in machine learning and artificial intelligence have paved the way for researchers in academia and industry to predict the specific material behaviors from sensor data and structural health monitoring [27,28,29,30,31]. Liu et al. incorporated the acoustic emission technique and K-means clustering method to identify damage modes in wind turbine blade composites [32,33]. Su et al. researched predicting FRP’s and concrete’s interfacial bond strength using different regression learning models [34]. Baghaei et al. used an artificial neural network to model open source data on FRP-to-concrete bonds to assess the durability under moisture conditions [35]. 

This current work is a first that provides a comprehensive data driven analysis to establish the connection between the moisture absorption in polymer composites and the dielectric response of the material. This article presents a two-part study. Firstly, quadratic discriminant analysis (QDA), support vector machines (SVM), and multilayer perceptron (MLP) classification models, accompanied by principal component analysis (PCA), were developed to classify the composites according to their current moisture state using the dielectric data. Then, multiple linear regression (MLR), decision tree regression (DTR), and multilayer perceptron (MLP) regression models were developed from dielectric data. The proposed models deliver a framework to accurately predict relative moisture absorption (M%) through a non-destructive technique, BbDS, which can be applied in real-life structures to monitor the absorbed moisture state using dielectric state variables. 

## 2. Materials and Methods

### 2.1. Material Preparation and Aging

Test specimens were created in this study using epoxy/glass fiber prepregs (manufacturer: Rock West Composites, Inc., San Diego, CA, USA). The reinforcement was unidirectional E-glass fiber, and the matrix was Propreg 250F, an epoxy-based thermoset resin. Three distinct unidirectional panels were created, each with four plies (Panel A), eight plies (Panel B), and twelve plies (Panel C). In this technique, the sequentially stacked prepregs were sandwiched between two aluminum plates with release film on both sides and placed into the compression molding chamber. The laminates were cured at 135 °C for 90 min under 300 kPa pressure. The laminates were cut into 19 mm × 250 mm rectangular coupons for aging and testing. The average thicknesses of Panels A, B, and C were 0.95 mm, 1.40 mm, and 1.95 mm, respectively.

### 2.2. Experimental Techniques

#### 2.2.1. Gravimetric Analysis

To begin, each dry sample was weighed using a precision scale with a 0.001 g accuracy before being submerged in 70 °C distilled water to imitate high humidity and wetness. The high temperature was used to accelerate the deterioration of the GFRP composite, in accordance with the ASTM D5229 method BWEP [36]. At regular intervals, the samples were weighed. To avoid the influence of surface water molecules, the samples were cleaned with a lint-free cloth and high-pressure air each time they were taken from the water. Then, using Equation (1), relative moisture absorption (M) was calculated.

(1) $$M\;\left(\%\right)=\frac{W_{t}{\;-\;W}_{o}}{W_{o}}\;\times \;100\%$$

where W_o_ is the initial mass of the dry sample, and W_t_ is the mass of moisture absorbed sample. Moisture intake measurements were carried out until the samples reached a saturated state, when the change in M was low and steady.

#### 2.2.2. Impedance Spectroscopy (IS)/Broadband Dielectric Spectroscopy (BBDS)

**Figure 4:**
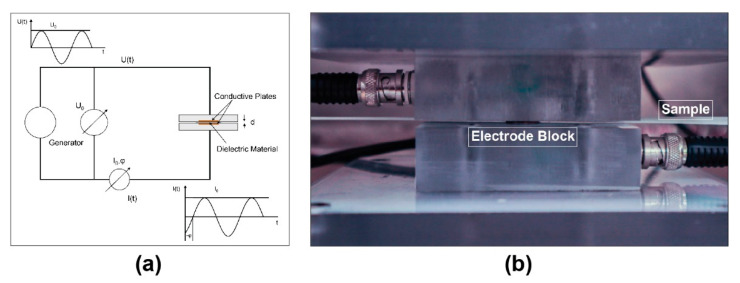
(**a**). Schematic of a sample placement between the electrodes in BbDS equipment [37]. (**b**). Experimental setup for BbDS.

Dielectric measurements were taken at regular intervals after weighing the samples in their initial state and after aging. The dielectric data was collected using a Novocontrol^®^ broadband dielectric spectrometer (manufacturer: Novocontrol Technologies GmbH & Co., Montabaur, Germany) in this investigation. An alpha analyzer is used in this machine to measure the complex dielectric value and impedance of the sample under examination as a function of frequency. This procedure holds the sample between two copper electrodes to ensure good contact (Figure 4). The experiments are conducted inside a faraday cage to eliminate electromagnetic interference. This setup resembles a simple parallel plate capacitor, since GFRP composites are dielectric. The analyzer applies a 1000 mV voltage across the sample at varied frequencies to determine the material’s dielectric characteristics. The measurements were obtained by a frequency sweep from 1 MHz to 1 Hz, with a scaling factor of 1.8.

### 2.3. Data Curation

#### 2.3.1. Dataset Preparation

As a first step in developing ML models, the raw data from the equipment has to be sorted. For the classification study, the moisture state of the samples was divided into three classes in dataset A. Table 1 shows the classes and their definition, as used to generate the dataset. Relative moisture absorption of 2.2% was taken as the limiting value, after what saturation was observed from the experiments.

Dielectric data was acquired from BBDS experiments, and M% was derived from gravimetric measurements. From the frequency sweep of every single specimen at three moisture states, real permittivity and dielectric relaxation strength (DRS) [20] values were stored. The thickness of the corresponding specimens, the real permittivity at 30 frequencies, and DRS were used as features for each data point, and they were labeled based on the moisture state of the individual. For regression model development, the accurate M% value was defined as the label for each observation in dataset B. In total, 228 sample data points were stored to prepare parent dataset A for classification. On the other hand, 130 observations were stored in dataset B for regression, which were collected at regular time intervals until saturation was achieved. The datasets were put into array X for features and vector y for the labels. These matrices later have been divided into training and testing sets using random splitting (80% for training, 20% for testing). The training datasets had been divided into training and validation folds for K-fold cross-validation, as described in Section 2.5. In summary, the structure of the datasets is shown in Table 2.

#### 2.3.2. Feature Scaling

As a general practice in machine learning, feature scaling was implemented in the parent dataset. Some of the classification algorithms (PCA, SVM) implemented in this study are distance-based computations. In this study, dielectric values at a lower frequency range have a broad range of values over the moisture absorption phases. If the features are not scaled, these particular features will govern the distance. Hence, the model output will not be the true picture of the dataset. So, Z-score standardization was selected as the feature scaling procedure in the classification algorithms. In standardization, the feature values are centered on the mean value (μ), with a unit standard deviation (σ). In this case, the mean of a specific attribute becomes zero, and the resultant distribution has a unit standard deviation. The formula of standardization is shown in Equation (2). On the other hand, for regression algorithms, the corresponding parent dataset was scaled using min–max normalization, as shown in Equation (3).

(2) $$z=\frac{\left(x\;-\;\mu \right)}{\sigma \;}$$

(3) $$x\prime =\frac{x\;-\;\min(x)}{\max\left(x\right)-\;\min(x)}$$

### 2.4. Predictive Models

This section describes the theoretical aspects of the ML models used in this work.

#### 2.4.1. Principal Component Analysis (PCA)

Principal component analysis (PCA) is a dimensionality reduction technique widely used in machine learning to transform a large number of features into a few principal components (PCs), which represent most, if not all, of the information of the parent dataset. PCA transforms the inter-correlated quantitative features to linearly uncorrelated multivariate data, which helps reduce or eliminate the curse of dimensionality [38]. Reducing feature size using PCA also increases the dataset’s interpretability, and the computational cost of model development is reduced significantly. Mathematically, each principal component is a linear combination of all standardized input features, which can be written as shown in Equation (4).

(4) $${\mathrm{PC}}_{n}{=\varphi }_{1n}x_{1\;}{+\varphi }_{2n}x_{2\;}{+\ldots +\varphi }_{\mathrm{pn}}x_{p}$$

where $\varphi_{1n}$, …, $\varphi_{\mathrm{pn}}$ are the loading of the nth PC. 

To find the PCs, at first, the covariance matrix of the input data set is calculated as

(5) $$\mathrm{COV}=\frac{1}{n}{\;\times \;X}^{t}\;\times \;X$$

Then, the eigenvectors and eigenvalues of the COV matrix are found and ordered in a descending form. Each eigenvector and eigenvalue here depict the loading and variance of the corresponding PC, respectively. To reduce dimensionality, first k PCs can be selected, of which cumulative variance can largely represent the character of the parent dataset. In this study, PCA was performed on the whole dataset before dividing it into folds using K-fold CV, so that the dataset was uniformly transformed.

#### 2.4.2. Quadratic Discriminant Analysis (QDA)

Discriminant analysis (DA) is a technique that transforms input features into a lower dimensional space and maximizes the ratio of inter-class and intra-class variance to gain maximum class separability. Though DA can be used to classify, as well as to reduce, dimensions, in this study, DA is used to classify the training folds and evaluate classification accuracy. In DA, a decision boundary is developed that separates different classes. Based on the type of decision boundaries, two different methods of DA are used, i.e., linear discriminant analysis (LDA) and quadratic discriminant analysis (QDA). In this study, a QDA model is developed to classify the observations based on their moisture state. As the name suggests, QDA provides a quadratic decision boundary to classify the observations. QDA is derived from simple probabilistic models, and class prediction can be acquired using Bayes’ rule. Here, the likelihood of a data vector (class conditional density) can be denoted by P(X|y=k), and the prior probability for class k (where k = 1, 2, …, K) can be denoted as $\pi_{k}$. Using this information, from Bayes’ rule, the posterior probability of a class being assigned to a data vector can be defined as follows

(6) $$P\left(y=k|X=x\right)=\frac{{P(X|y=k)\;\times \;\pi }_{k}}{\sum_{l=1}^{K}(P\left(X|y=l\right){\;\times \;\pi }_{l})}$$

Here, the likelihood is modeled as a multivariate Gaussian distribution, as shown in Equation (7).

(7) $$P(X|y=k)=\frac{1}{{\left(2\pi \right)}^{\frac{K}{2}}{\left|\sum_{k}\right|}^{\frac{1}{2}}}\exp(-\frac{1}{2}{\left({x\;-\;\mu }_{k}\right)}^{t}\sum_{k}{}^{-1}\left({x\;-\;\mu }_{k}\right))$$

In QDA, the log of the posterior, namely discriminant function (DF, $\delta_{k}\left(x\right)$), is calculated to find the decision boundary between two classes using Equation (8). In this case, the algorithm does not assume the same covariance ($\sum_{k})$ for all classes as LDA. From the DFs, a class for an observation is predicted using Equation (9).

(8) $$\delta_{k}\left(x\right)=-\frac{1}{2}\log\left(\sum_{k}\right)-\frac{1}{2}{\left({x\;-\;\mu }_{k}\right)}^{t}\sum_{k}{}^{-1}\left({x\;-\;\mu }_{k}\right){+\log\pi }_{k}$$

(9) $$G\left(x\right){\;=\;\mathrm{argmax}}_{k\;}\delta_{k}\left(x\right)$$

#### 2.4.3. Support Vector Machine (SVM)

Support vector machine (SVM) [39] is a supervised discriminative classifier algorithm that, given a training dataset, outputs an optimal hyperplane between different classes. Contrary to QDA, SVM is a non-probabilistic binary linear classifier, which transforms training data to high-dimensional space and performs linear regression (Equation (10)) to find the boundary and maximize the distance between two classes [35]. Testing data is then mapped into the same space, and the corresponding class is predicted based on which side of the decision boundary the data resides.

(10) $$f\left(x\right)=\phi {\left(x\right)}^{t}\omega +\beta$$

where $\phi \left(x\right)$ is a transformation matrix that maps the input to the high-dimensional space, and β is the model bias. Given the training vector $x_{i}\in R,i=1,\ldots ,n$ in two classes and vector $y\in {\left\{1.-1\right\}}^{n}$, SVM finds ω and β, such that the prediction given by $\mathrm{sign}\left(\phi {\left(x\right)}^{t}\omega +\beta \right)$ is correct for most samples. In this procedure, SVM solves a convex optimizing problem [35],

(11) $$\underset{\omega ,\beta ,\zeta }{\min}\frac{1}{2}\omega^{t}\omega +C\overset{n}{\underset{i=1}{\sum }}\zeta_{i}$$

where $\zeta_{i}$ are the slack variables, and C is the penalty term, namely the inverse regularization parameter, that controls the strength of the penalty applied to a prediction when a sample is misclassified or falls within the decision margins. Then, SVM utilizes a set of mathematical functions called a kernel. The kernel takes the input data and transforms it into a required form. Different SVM algorithms utilize different types of kernel functions, including linear, non-linear, radial basis function (RBF), sigmoid, etc. Finally, SVM predicts the class using the decision function defined in Equation (12).

(12) $$f\left(x,\alpha \right)=\overset{n}{\underset{i=1\in \mathrm{SV}}{\sum }}y_{i}\alpha_{i}K\left(x_{i},x\right)+\beta$$

where $\alpha_{i}$ are the Lagrangian coefficients from the dual problem solution, and K is the kernel used. Unlike QDA, SVM has a few hyperparameters like C, kernel, and decision boundary shape, which can be changed to tune SVM predictions.

SVM is a memory-efficient algorithm that uses a bunch of training points, namely support vectors, to develop decision boundaries. Though SVM is a binary classification technique, this can also be used for a multiclass dataset, such as the dataset of this study. In this case, SVM was applied to test data in a combination of two classes from a number of classes and classifies the data to the class that appeared the most in the combinations. This is called one-vs-one (ovo) calculation. On the other hand, If K SVMs are applied each time, comparing the Kth class to the remaining K-1 classes, the class that is predicted the most is assigned to the test vector. This is called one-vs-rest (ovr) classification.

#### 2.4.4. Multi-Layer Perceptron (MLP)

**Figure 5:**
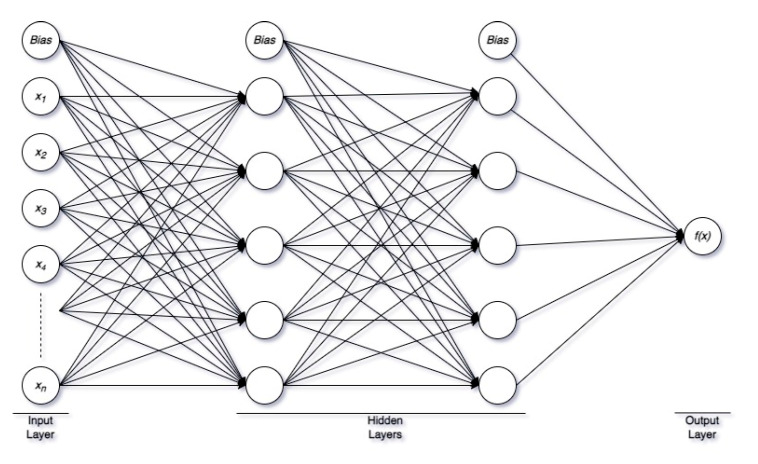
A schematic of a simple neural network used in MLP.

In this work, a fully connected, feed-forward artificial neural network (ANN), named multilayer perceptron (MLP) model, has been developed for classification and regression studies to predict the current moisture state in the composite. MLP is a supervised learning algorithm with at least three sequential layers, i.e., the input layer, hidden layer, and output layer. Each layer provides a set of output vectors that work as the input vector of the next layer. Figure 5 shows a simple MLP structure. The input layer consists of the raw input data with n features (also known as neurons/nodes). 

There can be more than one hidden layer based on the application. In MLP, each neuron in the hidden layer transforms the values from the previous layer with a weighted linear summation $w_{1}x_{1}+w_{2}x_{2}+\ldots +w_{n}x_{n}$, followed by a non-linear activation function. In a neural network, an activation function describes how the weighted sum of the input is turned into an output from a node or nodes in a layer. There are different activation functions used in MLP applications, i.e., logistic sigmoid function, hyperbolic tangent function, and rectified linear unit function (relu). All hidden layers typically use the same activation function. The output layer is simply the label (for classification) or the numerical target value (for regression), which it receives from the last hidden layer and transforms into proper output. 

#### 2.4.5. Multiple Linear Regression (MLR)

MLR is an extension of simple linear regression, which is used to predict the output of a variable (y ∈ **R**^m^^×^^1^), which is dependent on two or more independent variables or features (X_m,n_ ∈ **R**^m × n^). Equation (13) shows the MLR model, where $\omega_{1},\ldots ,\omega_{n}$ are the weights associated with corresponding feature vector X_n_, and $\omega_{0}$ is the y-intercept for x_0_ = 1. The outputs from the model, f(X_m_), are compared with the true y values to calculate the residual sum of squares (RSS) value using Equation (14). The weight vector ω is then achieved by minimizing the RSS value through Equation (15).

(13) $${f(X}_{m}{)=x}_{0}\omega_{0}{+x}_{m,1}\omega_{1}{+\ldots +x}_{m,n}\omega_{n}\;{=\omega }_{0}{+\omega }^{T}X_{m}$$

(14) $$\mathrm{RSS}(\omega )=\overset{m}{\underset{i=1}{\sum }}{{(y}_{i}\;-\;f\left(X_{i}\right))}^{2}$$

(15) $$\omega ={\left(X^{T}X\right)}^{-1}X^{T}y$$

#### 2.4.6. Decision Tree Regression (DTR)

**Figure 6:**
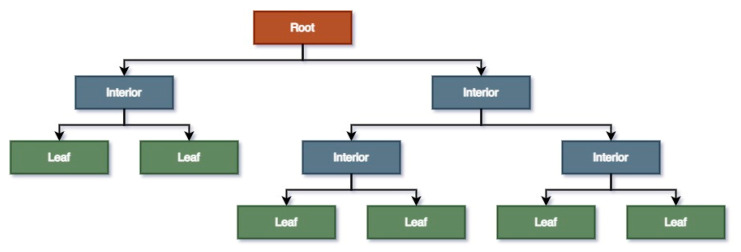
Terminology of a decision tree’s components with depth 3.

DTR is a very popular supervised learning method that was proposed by Breiman et al. [40]. DTR trains a model in the form of a tree to predict data and generate relevant continuous output by observing the features of an object. A decision tree has three types of nodes, i.e., root, interior, and leaf nodes. The root node represents the whole training sample, split into further nodes, namely the interior nodes. They provide the information from the features from the dataset, and their branches explain the decision conditions that generate the leaf nodes representing the outcome. A test data point in a DTR model starts at the root and progresses through the interior nodes, satisfying decision rules until it reaches a certain leaf. Finally, the average value of that leaf is selected as the output. Figure 6 shows a simple decision tree with depth 3. 

### 2.5. Hyperparameter Tuning and Cross-Validation

Most ML models have certain hyperparameters which the user may adjust before training the model, in contrast to model parameters, which are learned during model training and cannot be altered arbitrarily. These hyperparameters govern the accuracy of prediction and computational cost. In this work, the grid search (GS) technique has been used to tune the hyperparameters. GS reads a dictionary of predefined hyperparameters and reports back the best model evaluation parameter by developing and testing different models using a different combination of the given hyperparameters. 

**Figure 7:**
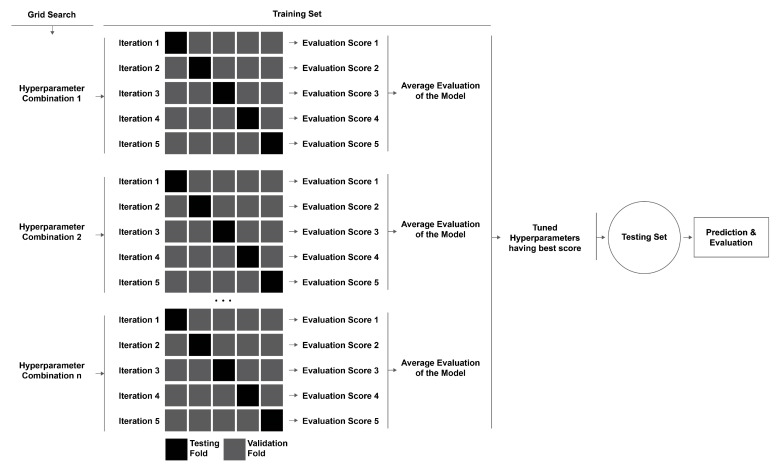
Hyperparameter tuning example in training set with 5-fold cross-validation, grid search, and final output prediction algorithm.

In this work, dataset A is limited to 228 observations, and dataset B is limited to 130 observations. So, a resampling procedure, named K-fold cross-validation, is used to evaluate the skill of the implemented machine learning models. In this method, the parent dataset is divided into two sets, the training and testing sets, and then the training set is split into K folds after shuffling the data points randomly. Then, for the K iteration, the Kth group is defined as the validation dataset, and the rest of the (K-1) datasets are used for training. The respective model is fitted to the training dataset in each iteration and evaluated using the validation dataset. The evaluation parameters are then retained, and the model is discarded. After the K iterations, an average evaluation score of all models is returned, which summarizes the model’s skill on the whole dataset. In this method, each sample data point is allowed to be used in the validation dataset once and used to train the model K-1 times. In this study, GS and K-fold cross-validation are combined (Figure 7). For each combination of hyperparameters from GS, K-fold cross-validation has been performed to find the most accurate combination of hyperparameters to develop the model. Then, the model is used to predict the outcomes of the previously held test dataset. 

### 2.6. Prediction Parameter Definitions

In this section the following parameters have been reported for the developed models to evaluate and compare the performance of the models on test dataset.

- Classification accuracy

(16) $$\mathrm{Accuracy}=\frac{\mathrm{number}\;\mathrm{of}\;\mathrm{correctly}\;\mathrm{predicted}\;\mathrm{data}\;\mathrm{points}}{\mathrm{total}\;\mathrm{data}\;\mathrm{points}\;\mathrm{in}\;\mathrm{the}\;\mathrm{test}\;\mathrm{dataset}}$$

- Precision

(17) $$\mathrm{Precision}=\frac{\mathrm{number}\;\mathrm{of}\;\mathrm{correctly}\;\mathrm{predicted}\;\mathrm{positive}\;\mathrm{instances}}{\mathrm{number}\;\mathrm{of}\;\mathrm{total}\;\mathrm{positive}\;\mathrm{predictions}}$$

- Recall

(18) $$\mathrm{Recall}=\frac{\mathrm{number}\;\mathrm{of}\;\mathrm{correctly}\;\mathrm{predicted}\;\mathrm{positive}\;\mathrm{instances}}{\mathrm{number}\;\mathrm{of}\;\mathrm{total}\;\mathrm{relavent}\;\mathrm{instances}}$$

- F1-Score

(19) $$F1-\mathrm{Score}=2\;\times \;\frac{\mathrm{Precision}\times \mathrm{Recall}}{\mathrm{Precision}+\mathrm{Recall}}$$

- Coefficient of determination: R^2^ score

(20) $$R^{2}=1\;-\;\frac{\sum_{i}{\left(\mathrm{true}\;\mathrm{value}-\mathrm{predicted}\;\mathrm{value}\right)}^{2}}{\sum_{i}{\left(\mathrm{true}\;\mathrm{value}-\mathrm{mean}\right)}^{2}}$$

- Mean squared error (MSE)

(21) $$\mathrm{MSE}=\frac{1}{\mathrm{number}\;\mathrm{of}\;\mathrm{samples}\;(N)}\overset{N}{\underset{i=1}{\sum }}{\left(\mathrm{true}\;\mathrm{value}-\mathrm{predicted}\;\mathrm{value}\right)}^{2}$$

- Mean absolute error (MAE)

(22) $$\mathrm{MAE}=\frac{1}{\mathrm{number}\;\mathrm{of}\;\mathrm{samples}\;(N)}\overset{N}{\underset{i=1}{\sum }}\left|\mathrm{true}\;\mathrm{value}-\mathrm{predicted}\;\mathrm{value}\right|$$

## 3. Model Development and Discussion

### 3.1. Classification Algorithms

#### 3.1.1. Dimensionality and Multicollinearity Reduction

**Figure 8:**
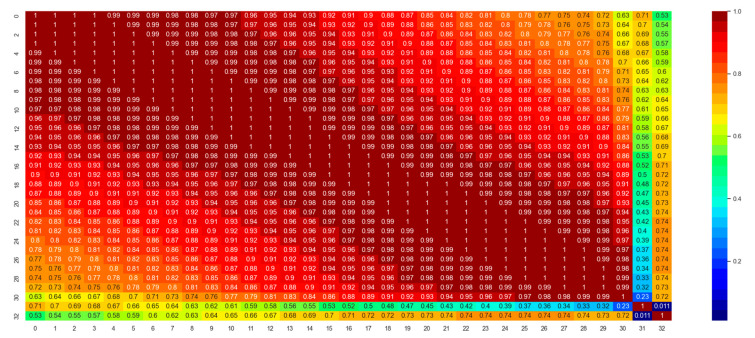
Correlation matrix for dataset A. Here, columns indexed by from 0 to 31 are the features, and the column with index 32 contains the labels.

The first important thing to develop a machine learning model is to understand the dataset and feature characteristics. Figure 8 shows the correlation matrix of the features and the label for the classification dataset. Correlation is a statistical parameter that represents to which extent multiple variables fluctuate in tandem [41]. A high correlation between the independent and dependent variables implies that the independent variable has high significance in governing the output. However, a high correlation between two different independent variables implies that the two variables are redundant, which is undesired in ML model development. This leads to computational inefficiency and memory wastage. Here, columns indexed by from 0 to 31 are the features, and the column with index 32 contains the labels. From Figure 8, it can be seen that the correlation between the features and labels are high enough (>0.5), from a statistics point of view. However, the thickness feature (index 31) is not very well-correlated (0.011 < 0.5) with the data labels. This means, unlike the dielectric permittivity features, the thickness feature does not affect the output labels significantly.

**Figure 9:**
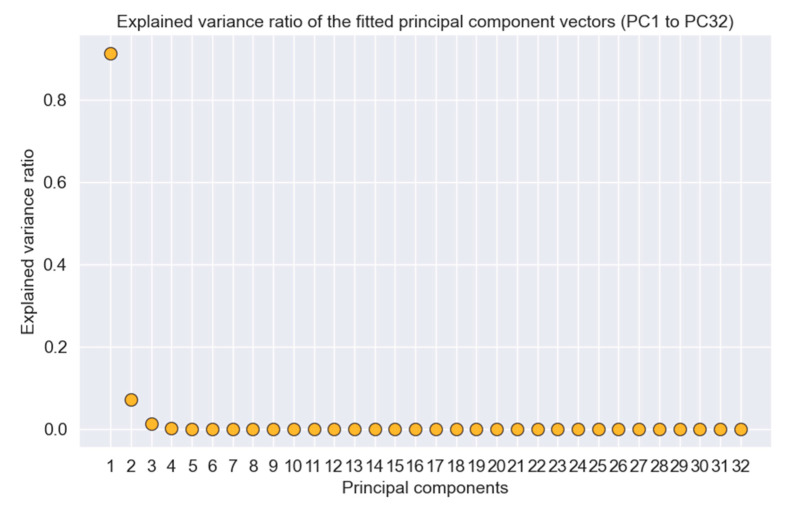
Explained variance ratio for 32 principal components.

**Figure 10:**
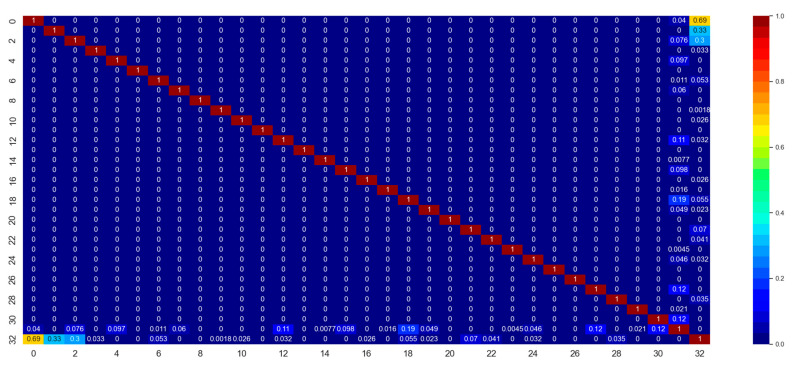
Correlation matrix for the parent dataset A after PCA transformation.

Figure 8 also indicates that the features are inter-correlated. This phenomenon is called data multicollinearity (DM) [42]. This correlation between the ‘independent’ features can cause a problem in ML model development and provide unreliable, erroneous data, as they are supposed to be ‘independent’. Because of DM, coefficients for one variable can change significantly because of other independent variables, and it can cause the coefficients to be very sensitive to small changes in the model. PCA has been implemented in the dataset to eliminate this multicollinearity problem. PCA resolves this problem by transforming inter-correlated multivariate data to linearly uncorrelated multivariate data. PCA also reduces the dimension, which, in turn, saves computational costs. Figure 9 shows the explained variance ratio for 32 principal components (PCs). The first three PCs’ cumulative explained variance ratios were nearly 1.0, which implies that these three PCs are enough to describe the information provided by the entire dataset. Thus, PCA reduces the dimension from 32 to 3, which reduces the required computational time for the classification and regression models. After PCA transformation, the correlation matrix for the updated dataset was acquired (Figure 10), where it was clearly visible that there was no correlation between the features. Moreover, PC-1 itself had the maximum correlation with the output 0.69, implying the most significant PC on the output. PC-2 and PC-3 had correlation coefficients of 0.33 and 0.30, which, though less than 0.5, have also been used in this study to develop the models to the highest accuracy possible.

#### 3.1.2. Performance Evaluation

After data sorting and data curation, the K-fold cross-validation technique was adopted in the training set. However, the number of folds to have maximum accuracy and minimum variance needs to be determined through a K-fold sensitivity analysis. In this study, the parent dataset was run through multiple iterations by changing the number of folds to find the optimum value for K for this dataset. Through this analysis, it was found that, for K = 9, each model shows the maximum accuracy with minimum variance. Hence, K = 9 was selected for the cross-validation of the models.

In this study, the composite specimens were divided into three classes, on the basis of their relative moisture absorption (M%). The real permittivity values in 30 frequencies, DRS value, and thickness were the 32 features that were transformed into three PCs. Then, the dataset was randomly divided into two sets: the training set (80%) and the testing set (20%). The training dataset was cross-validated for different hyperparameters of different models using a 9-fold validation technique. In this study, the hyperparameters for the SVM and MLP classifiers were determined through a tuning process. QDA does not need any tuning, as QDA does not have any hyperparameters. The optimal hyperparameters of these algorithms have been tabulated in Table 3. There are three hyperparameters to tune for SVM to get a balance of more accuracy and computational cost and avoid overfitting, i.e., inverse regularization parameter (C), kernel function, and decision function shape. C in SVM dictates the penalty of misclassification and the width of the margin. A high C means a low margin, which ensures a high penalty for misclassification and vice versa. An increase in C also decreases the bias and increases the variance. Seemingly, a high C value may perform well in the training set, but in unseen test data, it may not perform well, as a high C can overfit the data. So, a C value must be tuned in such a way that an optimum value is obtained for a bias–variance trade-off. In this study, radial basis function (RBF) [43] was selected as the kernel function after tuning the related hyperparameters. RBF mostly performs well when the features have a non-linear relationship with the output label, as a decision function shape one-vs-one approach was selected.

On the other hand, for the MLP classifier, the activation function and number of hidden layers and sizes were most crucial for an accurate, yet less computationally exhaustive, analysis. In this work, a logistic function was used as the activation function. Here, a number of hidden layers and sizes were obtained using a grid search. 

For performance validation, the three models, QDA, SVM, and MLP, were developed on the parent dataset, and PCA transformed the dataset to find the mean accuracy of the specific models for the 9-fold, cross-validated and test dataset. 

The summary of the mean validation and test accuracies for the three algorithms is shown in Table 4. By reducing dimensions and eliminating multicollinearity using PCA, it can be seen that the mean accuracy of the validation sets for all three models increases. However, QDA performs way better when PCA is used beforehand on the dataset, as multicollinearity is resolved. Without using PCA, the accuracy was only 67.3% (the lowest of them all), but using PCA, the mean accuracy climbed to 96.17%. Furthermore, the use of PCA has a similarly significant effect on the prediction accuracy of the test dataset, in terms of applying QDA. With PCA, QDA shows a 10.88% increase in prediction accuracy, which is similar to the more sophisticated ML model SVM’s accuracy. However, using PCA does not have an effect on the SVM and MLP classifier’s prediction accuracy. It means that the effect of high dimensionality and multicollinearity does not significantly affect SVM and MLP. Nevertheless, it reduced computational costs for SVM significantly (50%), and for MLP, in a reduced manner (6.84%). So, the use of PCA is particularly important when the dataset is comparatively larger and more complex. Not to mention, the overall comparison implies that MLP shows a better accuracy (97.83%) in predicting the current moisture state of the composite from dielectric permittivity than SVM (93.48%). However, the runtime for MLP is almost 70 times higher than SVM. This is due to the complex interlayer computations, which take much more time than the SVM algorithm.

**Figure 11:**
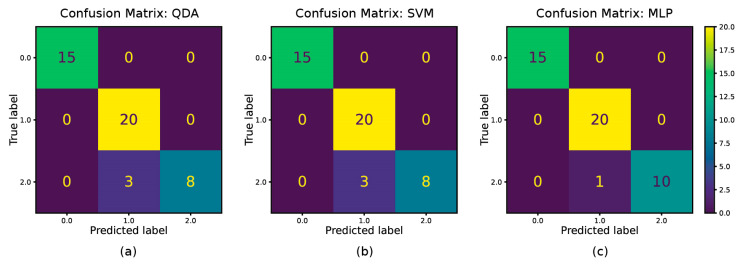
Confusion matrices for (**a**). QDA (with PCA), (**b**). SVM, and (**c**). MLP classifiers.

Figure 11 shows the confusion matrices for the developed models applied to a testing dataset to predict whether the individual samples were either dry (label 0) or moisture absorbed but not saturated (label 1) or moisture saturated (label 2). Table 5 further tabulates the class-wise precision, recall, and F1 scores. It can be seen from Figure 11 and Table 5 that all of the three models can predict the dry state, with 100% of accuracy (F1 score 1.00 for class 0). However, they get confused with the saturated and non-saturated states. MLP does quite a good job having only one wrong prediction (F1 score 0.98 for class 1 and 0.95 for class 2), while SVM and QDA (with PCA) share similar results, having more wrong outputs (F1 score 0.93 for class 1 and 0.84 for class 2). 

Here, to conclude, SVM, MLP, and QDA (along with PCA) provide versatile models that can accurately predict the composite’s moisture state from the dielectric parameters, even if the features are intercorrelated. However, MLP’s computational cost is significantly higher than the other models. It makes SVM and QDA (with PCA) the best-performing models in this study.

### 3.2. Regression Algorithms

#### Development and Performance Evaluation

This section reports the accuracy and efficiency of the regression models used in this study. Regression study is particularly important, in the context of classification algorithms, which can only separate specimens, in terms of the amount of moisture present in the specimen, whereas regression can predict a continuous quantity. Three different models have been developed in this study—MLR, DTR, and MLP regressors. These models have been compared here, in terms of their performance indicators, such as the R^2^ value (defined in A.1). These models can predict the numerical values of relative moisture absorption (M%) from the dielectric permittivity data. At first, dataset B was normalized and divided into two sets randomly, namely the training set (80%) and the testing set (20%). The models were validated using the K-fold cross-validation technique. A K-fold sensitivity analysis for the regression models was also performed, and the value of K was selected as 10 for its high R^2^ value and low variance. The results of the respective models are discussed in the next part. 

**Figure 12:**
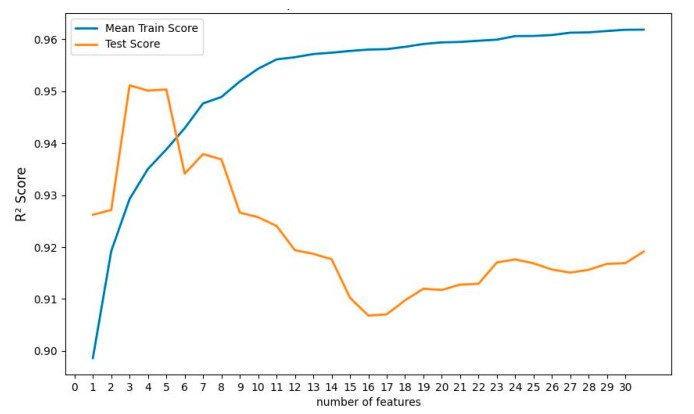
R^2^ score for the training set and testing set with the different number of features (ranked) used in MLR.

Multiple linear regression (MLR) has no hyperparameters to tune. However, the output can vary, depending on how many features it uses. For example, a feature that has no correlation with the output can actually do more harm to the model, rather than developing it. So, the recursive feature elimination (RFE) technique was adapted to find which features in the training folds were most effective in predicting the target. At first, RFE fits the MLR model with all of the features and ranks the importance of the features. In the next step, RFE discards the least important features and re-fits the model to evaluate the performance. This step is performed until a desired number of features is reached. In this work, RFE was performed to fit and test the MLR model, with 1–32 features in different subsets. The results are shown in Figure 12. The figure shows that using only the first three important features gives the best performance of the model. The first three important features are real permittivity values at 542 Hz, 339 Hz, and 1390 Hz. If the MLR model is developed using the real permittivity values at these frequencies, the coefficient of determination R^2^ of the predicted and true test data is 0.9511.

**Figure 13:**
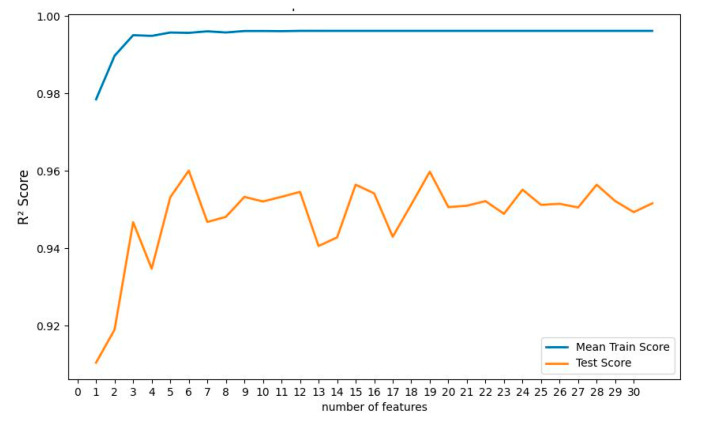
R^2^ score for the training set and testing set with the different number of features (ranked) used in DTR.

The decision tree regressor (DTR) model has also been developed using 10-Fold cross-validation and the RFE technique. The maximum depth of the tree was nine, and the mean squared error (MSE) function was selected to measure the quality of the split in the internal nodes. MSE function minimizes the L2 loss using the mean of each terminal node. These hyperparameters were obtained using the grid search technique. Figure 13 shows the R^2^ score for the RFE analysis of the DTR model. It can be seen that the maximum R^2^ value (0.9605) achieved from the DTR model was when the first six most important features were selected to develop the model. The corresponding features include real permittivity values at 212 Hz, 2 Hz, 52 Hz, 827 Hz, 1388 Hz, and DRS values. However, using all the features does not harm the model output as severely as in the case of MLR. If all 32 of the features are used to develop the model, the R^2^ value of the model on the test data is 0.9544, which is very close to the maximum obtained R^2^ value of 0.9605.

In this work, an artificial neural network-based regressor multi-layer perceptron (MLP) has also been developed to estimate M from dielectric data. For a good performance of the model, exhaustive hyperparameter tuning has been performed. The crucial hyperparameters for MLP regressor are the activation function and hidden layer number and size. A total of 2,307,432 models have been developed using a combination of three hidden layers and 10–100 nodes per hidden layer. It was found that, with three hidden layers, with the following combination of (96, 88, 31) and hyperbolic tangent activation function, the best R^2^ value of 0.9620 was obtained. 

Apart from the accuracy of the estimation, the computational cost is an important aspect of developing regression models. Though the MLP regressor provides a slightly better R^2^ score than the two other models, it takes almost three times more computational time than MLR and almost two times more computational time than DTR. In summary, all three developed models can accurately predict the relative moisture absorption value, with an R^2^ value of more than 0.95. The summary of the model performance metrics has been tabulated in Table 6.

### 3.3. Model Interpretation Complying Physics

Prediction accuracy and optimized computational cost are two important aspects of any ML algorithm. However, due to the black-box nature of the algorithms, it is hardly interpretable to the users. In this work, dielectric characteristics of composite specimens under hygrothermal loading are used to predict the current moisture state and estimate the relative moisture absorption. In the previous sections, the accuracy and efficiency of the algorithms are explained, but one question comes into prospect—which features governed the outputs?

Feature permutation importance (PI) is a feature inspection method that estimates the feature importance, based on the impact of an individual feature on the model’s outputs. In this method, to test a feature’s significance, the observations of the corresponding features are shuffled, and the accuracy of the fitted modified dataset is compared with the fitted parent dataset. If shuffling a particular column changes the prediction error, the difference in the metric is assigned to the feature as PI. Features with high PI mean they are significant in predicting the model’s outputs. 

**Figure 14:**
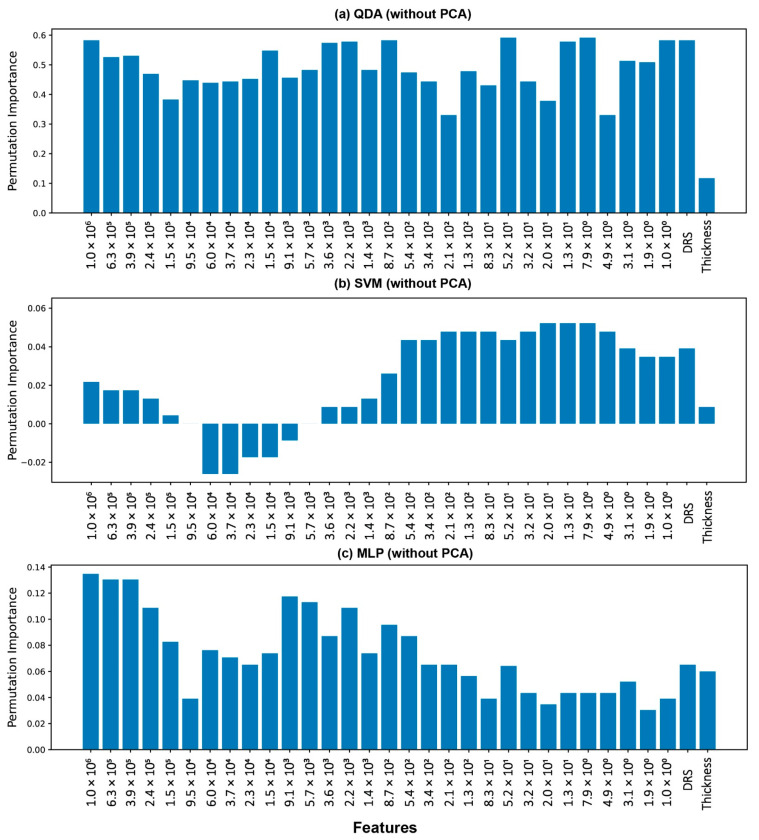
Permutation importance (PI) values for features used in developing (**a**). QDA, (**b**). SVM, and (**c**). MLP, without PCA.

Figure 14 shows the PI values for each feature in the three classification models (QDA, SVM, MLP). PCA was not performed to find the PI on the QDA model, as PCA reduces the dimension. From the figure, for QDA, the PI for each feature was very close. This is due to the fact that the features were intercorrelated, so each feature was somewhat involved in predicting the output. However, the accuracy of this model (QDA without PCA) was only 67.3%. This model was not successful in predicting the outputs with great accuracy, and this was not a representation of the physics behind the moisture absorption phenomenon. So, these PI values cannot be interpreted to learn the physics. Now, for SVM, the real permittivity features, in the range of 1 to 540 Hz, and DRS show the maximum PI values, compared to the permittivity at other frequencies and thickness features. This result accurately proves the underpinning physics here. In composites, water molecules can reside in two distinct forms—free and bound. As water molecules are inherently dipolar, they deploy a dipolar contribution to the real permittivity, which is observable around 1000 Hz for most materials. Secondly, since it is vulnerable to the concentration of polar molecule components, the permittivity response is especially sensitive to changes in a material’s chemical structure. Water molecules attach to the hydrophilic groups on the polymer chain during moisture absorption, altering the polymer’s mobility. Additionally, as a result of the plasticization effect, it also changes molecular relaxation. This phenomenon is also clearly seen in the lower frequency range (<1000 Hz). Last, but not least, ionic contributions are seen below 10 Hz when a material’s real permittivity increases as its conductivity increases. So, the SVM algorithm can pick those patterns in the permittivity data, which is governed by distinct physics. However, in the case of the MLP classifier model, there was no pattern in the PI values for the features. This might be due to the fact that, in MLP, each feature is interconnected to the nodes in the hidden layer, and their correlated effects predict the model output. Even if one feature is shuffled, the model struggles to predict the output accurately.

To interpret the results of the regression models, the recursive feature elimination (RFE) technique was adopted. In RFE, features are ranked by importance using the model-dependent feature importance method. For instance, in multiple linear regression (MLR), regression coefficients are used to rank the features. In the developed MLR model, the output is governed by the real permittivity values at 542 Hz, 339 Hz, and 1390 Hz. On the other hand, in DTR, the importance of a feature is determined as the normalized total reduction of the R^2^ score brought by the feature. This is called GINI importance [44]. RFE utilizes this parameter to rank the features in the case of DTR. In this study, the following six features (real permittivity values at 212 Hz, 2 Hz, 52 Hz, 827 Hz, 1390 Hz, and DRS value) are the most important features, which can estimate the relative moisture absorption for a given sample, with an R^2^ value of 0.9605. So, MLR and DTR are mostly governed by real permittivity values near or under 1000 Hz. This can be attributed to the dipolar polarization that is observable near 1000 Hz. This effect is a direct indicator of the moisture absorption phenomenon, as reported in the literature [16,45]. 

Not to mention, the models developed in this study are not universal to all configurations and material systems of all polymer composite structures. However, this work opens the scope, in order to develop a global data-driven machine learning model that can estimate the moisture content in a variety of material systems exposed to varied environmental factors. To achieve this feat, the work has to be expanded to acquire new data samples, including more variability in the materials system and aging parameters. This application can also be expanded to predict the mechanical strength degradation from the dielectric data of polymer composites under hygrothermal loading, which is also a scope of future study.

## 4. Conclusions

This work has provided, for the first time, a framework incorporating different machine learning algorithms and dielectric responses from polymer composites under hygrothermal aging to predict the moisture content accurately. Classification models, i.e., quadratic discriminant analysis (QDA), support vector machine (SVM), and multilayer perceptron (MLP), and regression models, i.e., multiple linear regression (MLR), decision tree regressor (DTR), and multiplayer perceptron (MLP), were developed to quantitively estimate the relative moisture absorption (M%) of the composites. Finally, permutation importance (PI) and recursive feature elimination (RFE) techniques were adopted to understand the dielectric response at which frequencies govern the model’s prediction accuracy. The following conclusions can be drawn from this study:

- QDA with multicollinearity reduction using principal component analysis (PCA), SVM, and MLP—each provide effective models that can predict the saturation state of the composite with accuracies of 93.48%, 93.48%, and 97.83%, respectively.
- Developed MLR, DTR, and MLP regression models can estimate M% from dielectric state variables, with R^2^ scores of 0.9511, 0.9605, and 0.9620, respectively.
- The PI values indicate that real permittivity values in the range of 1 to 540 Hz and dielectric relaxation strength (DRS) mostly dictate the classification models’ higher accuracies, whereas the RFE values indicate that the real permittivity values in the range of 1 Hz to 1390 Hz mostly dictate the high R^2^ values for regression models. This can be attributed to the interfacial polarization, dipolar polarization, and plasticization phenomena that come into perspective, due to moisture absorption.

To conclude, with tremendous accuracy, the developed models can predict a test sample’s material state changes, due to the hygrothermal effects using dielectric state variables. Consequently, a global data-driven model can be developed and implemented in real-life structural health monitoring.

## Figures and Tables

**Table 1:** Definitions of classes used.

Class Label	Class Name	Relative Moisture Absorption (M%)	Description
0	Dry	0%	No moisture absorbed
1	Non-saturated	0% < M < 2.2%	Moisture absorbed, but not saturated
2	Saturated	M ≥ 2.2%	Moisture saturated

**Table 2:** Structure of the datasets.

Datum	Features (32), X						Label, y
	f_1_	f_2_	*……………………*	f_30_	DRS	Thickness	
1	x_1,1_	x_1,2_	……………………	x_1,30_	x_1,31_	x_1,32_	y_1_
:	:	:	:	:	:	:	:
:	:	:	:	:	:	:	:
n	x_n,1_	x_n,2_	……………………	x_n,30_	x_n,31_	x_n,32_	y_n_

**Table 3:** Tuned hyperparameters for classification models.

Model	Parameter	Value
SVM	Inverse regularization parameter (C)	5
	Kernel function	RBF (radial basis function)
	Decision function shape	‘ovo’ (one-vs-one)
MLP	Hidden layer sizes	(10)
	Activation function	Logistic
	Maximum iteration	500

**Table 4:** Performance summary for validation and test set.

Model	Validation Set			Test Set		
	Without PCA	With PCA	Change	Without PCA	With PCA	Change
QDA	67.3%	96.17%	+28.87%	82.6%	93.48%	+10.88%
	Min. 45.0% Max 80.95%	Min. 90.48% Max 100.00%		Runtime: 0.001 s	Runtime: 0.00099 s	−1%
SVM	92.77%	93.89%	+1.12%	93.48%	93.48%	+0%
	Min. 85.00% Max 100.00%	Min. 85.00% Max 100.00%		Runtime: 0.002 s	Runtime: 0.001 s	−50%
MLP	98.33%	98.90%	+0.0.57%	97.83%	97.83%	0%
	Min. 95.00% Max 100.00%	Min. 95.00% Max 100.00%		Runtime: 0.73 s	Runtime: 0.68 s	−6.84%

**Table 5:** Test data classification report summary for QDA (with PCA), SVM, and MLP classifiers.

Model	Class	Precision	Recall	F1-Score
QDA (with PCA)	0	1.00	1.00	1.00
	1	0.87	1.00	0.93
	2	1.00	0.73	0.84
SVM (without PCA)	0	1.00	1.00	1.00
	1	0.87	1.00	0.93
	2	1.00	0.73	0.84
MLP (without PCA)	0	1.00	1.00	1.00
	1	0.95	1.00	0.98
	2	1.00	0.91	0.95

**Table 6:** Tuned hyperparameters and performance parameters for MLR, DTR, and MLP regressors.

Model	Hyperparameters	R^2^	MSE	MAE	Computational Time (s)
MLR	Features to use: 3	0.9511	0.0408	0.1661	0.013
DTR	Features to use: 6 Maximum depth: 9	0.9605	0.0364	0.1248	0.019
MLP	Activation Function: tanh Hidden layer sizes: (96, 88, 31)	0.9620	0.0317	0.1466	0.041

## Data Availability

Data is available at corresponding authors.
